# First-trimester urinary glycolic acid and methylsuccinic acid measurement for preeclampsia prediction: A Systematic review protocol

**DOI:** 10.1371/journal.pone.0356735

**Published:** 2026-09-25

**Authors:** Svitsai Chagonda, Vitaris Kodogo, Fortunate Katambarare, Rumbidzai Mawira, Mandy Marume, Justen Manasa, Donald Tanyanyiwa, Jonathan Matenga

**Affiliations:** 1 Department of Laboratory Diagnostic and Investigative Sciences, University of Zimbabwe Faculty of Medicine and Health Sciences, Harare, Zimbabwe; 2 Department of Internal Medicine, University of Zimbabwe Faculty of Medicine and Health Sciences, Harare, Zimbabwe; Hokkaido University: Hokkaido Daigaku, JAPAN

## Abstract

**Introduction:**

Preeclampsia is an important cause of maternal and neonatal morbidity and mortality around the world, with hypertension on the rise in sub-Saharan Africa. First-trimester screening for preeclampsia is vital for guiding preventative therapies like low dose aspirin. Traditional biomarkers face accessibility barriers in low-resource settings. Urinary glycolic acid (GA) and methylsuccinic acid (MSA) have emerged as stable, cost-effective, non-invasive metabolic candidates. However, their prognostic value has not been systematically evaluated. This systematic review aims to evaluate the prognostic value of first trimester urinary glycolic acid and methylsuccinic acid for development of preeclampsia.

**Methods:**

This systematic review will be guided by the Preferred Reporting Items for Systematic Reviews and Meta-Analysis – Protocols (PRISMA-P) checklist and has been registered with PROSPERO (CRD420251057596). The following databases will be searched: Cochrane Library, Embase, PubMed/MEDLINE, Scopus, Web of Science, grey literature and trial registries from 2008 to August 2026. Eligible studies will include pregnant women in the first trimester (<14 weeks) with urinary GA and/or MSA measured, comparing those who develop preeclampsia to normotensive controls. Two independent reviewers will screen titles/abstracts and full texts, with conflicts resolved by a third reviewer. Risk of bias will be assessed using the Quality in Prognosis Studies (QUIPS) tool. Data will be synthesized narratively: if more than 3 studies report comparable metrics, a meta-analysis using a random effects model will be performed.

**Ethics and dissemination:**

As a secondary analysis of peer reviewed articles, this review does not require ethical approval. Findings from the review will be disseminated through conference presentations and peer-reviewed publication.

## Introduction

Preeclampsia (PE) is a hypertensive disorder of pregnancy that poses significant risks to maternal and neonatal health [1] and affects 2–8% of pregnancies, contributing to 10%–15% of maternal deaths and 15%–20% of preterm births worldwide [2]. It is believed that PE occurs in two stages, the first of which consists of shallow cytotrophoblast invasion of the maternal uterus. This shallow invasion leaves the spiral arteries rigid and narrow, thus leading to the second stage of placental ischemia and the release of anti-angiogenic proteins [3]. These proteins include soluble fms-like tyrosine kinase-1 (sFlt-1), which is a good predictor of PE when measured along with the angiogenic placental growth factor (PIGF) in the first trimester [4]. While the sFlt-1/PIGF ratio allows for identification and risk stratification of PE development, their accessibility and use are limited in low-resource settings [5].

Metabolomic profiling has identified urinary organic acids as alternative, non-invasive screening targets [6]. Although numerous candidate biomarkers for PE have been identified, glycolic acid (GA) and methylsuccinic acid (MSA) are promising prognostic factors, given their association with oxidative stress pathways implicated in defective placentation [7]. Biologically, GA is as a key indicator of peroxisomal metabolism, while MSA reflects mitochondrial fatty acid oxidation pathways. Dysregulation in these markers point to upstream mechanisms of placental oxidative stress, mitochondrial distress, and hypoxic trophoblast injury, which are foundational to preeclampsia pathogenesis [8]. Their small molecular size and stability in urine make them ideal candidates for lower-cost assays compared to PIGF, potentially enabling adoption in low-resource settings for first-trimester risk stratification. While studies have identified GA and MSA as elevated in the first trimester of pregnancies which develop preeclampsia, their prognostic value is yet to be explored. This review aims to synthesize evidence to evaluate the possibility of using urinary GA and MSA in the first trimester as non -invasive prognostic factors for preeclampsia development. That is, to evaluate their prognostic value and clinical potential.

A broad body of evidence has evaluated general urinary and untargeted metabolomic profiles in pregnancy-as synthesized in recent overviews. There remains a distinct gap regarding the isolated prognosis value of GA and MSA. Prior reviews encompass cross-trimester data or broad-spectrum untargeted platforms [9]. This review addresses this gap by isolating these two specific metabolites within the early intervention window to determine their clinical utility as a targeted risk-stratification tool. First trimester identification of those at high risk of developing PE allows for timely initiation of aspirin prophylaxis, which is most effective when initiated before 16 weeks gestation [10].

**Research question**: In pregnant women during their first trimester, what is the prognostic value of urinary of glycolic acid and/or methylsuccinic acid for subsequent development of preeclampsia?

**Primary Objective:** To evaluate the prognostic value of first-trimester urinary glycolic acid and methylsuccinic acid for subsequent development of preeclampsia.

## Methods

### Anticipated search start/end dates

Start- 10 December 2025

End – 31 August 2026

### Registration and reporting guideline

The systematic review protocol was developed following the Preferred Reporting Items for Systematic Reviews and Meta-Analysis Protocols (PRISMA-P) checklist [11]. The review was registered on PROSPERO (CRD420251057596). The completed review will be reported in accordance with the PRISMA statement.

### Eligibility criteria

#### Inclusion criteria.

Population: Pregnant women in the first trimester (< 14 weeks) all risk levels.

Index Factor: Measured urinary glycolic acid and/or methylsuccinic acid in the first trimester.

Comparator: Normotensive pregnant controls.

Outcome: development of preeclampsia including any subtypes.

Timing: GA and MSA measurement in the first trimester and follow-up for the outcome until delivery.

Setting: Antenatal care settings.

Articles published in English or with an available English translation.

#### Exclusion criteria.

Studies not performed on human subjects.

Articles where free full text is not available. *

*If full text of an otherwise eligible study is not accessible through institutional subscriptions, a request for the article may be made via interlibrary loan or by contacting the author directly via email. Complete unavailability will be documented as a limitation; however, studies will not be automatically excluded without retrieval attempt.

### Information sources

The following databases will be searched: Cochrane Library, Embase, PubMed/MEDLINE, Scopus, Web of Science. To optimise retrieval and lower potential publication bias, searches will be extended to include grey literature databases and international clinical trial registries. The reference lists of all included studies will additionally be hand-searched to capture any missed relevant literature.

### Patient and public involvement (PPI) statement

Patients or the public were not involved in the design, conduct, reporting, or dissemination plans of this systematic review protocol. As this is a protocol for a systematic review direct patient involvement was not feasible at this stage. Any patient-relevant outcomes identified will be communicated in accessible formats to ensure wider reach and impact.

### Search strategy

The following search terms (Table 1.) will be adapted to search each aforementioned information source.

**Table 1:** Search terms (PubMed).

Keyword	Alternatives
Pregnancy (P)	Pregnancy OR Pregnant Women OR expectant mother OR antepartum OR impregnation OR conceive OR Pregnant OR maternity OR conception OR foetation OR pregnancies OR gestation OR pregnancy trimester, first trimester
AND Glycolic acid (I) OR Methylsuccinic acid (I)	Glycolic acid OR hydroxyacetic acid OR 2-hydroxyethanoic acid OR 2-hydroxyacetic acid OR biomarkers OR metabolomics OR organic acids OR metabolite Methylsuccinic acid OR 2-methylsuccinic acid OR 2 -methylbutanedioic acid OR pyrotartaric acid OR 1,2 propanedicarboxylic acid OR methylbutane-1,4-dioic acid
AND Preeclampsia (O)	Preeclampsia OR Gestational hypertension OR Pregnancy induced hypertension OR Eclampsia OR Hypertension in Pregnancy OR Hypertensive Disorders of Pregnancy OR HELLP syndrome
Filtering	2008–2026, English language, Full text, Free Full text, Human, Female

The search filter captures literature published from 2008 to 2026. This temporal boundary is justified by the timeline of clinical technology deployment: 2008–2026 marks the period during which high-resolution mass spectrometry and standardized, reproducible metabolomic platforms capable of precisely quantifying specific organic acids (such as GA and MSA) became widely integrated into cohort-based clinical biomarker discovery [12].

### Study selection process

Records from databases will be collated in Zotero, deduplicated, and uploaded to Rayyan for screening [13,14]. Two reviewers will independently screen titles/abstracts and full texts, with conflicts resolved by a third reviewer. Selected abstracts will be followed by a full text review and creation of the PRISMA flow diagram. Data will be extracted using a pre-piloted form in Excel, stored securely on encrypted cloud servers, and analyzed in STATA and R [15]. All steps will adhere to PRISMA guidelines [16]. Cohen’s kappa (k) will be used to evaluate agreement between the reviewers [17].

Publications will be limited to those whose full text is available and were published in English or have an English translation available. The search strategy and all identified keywords/index terms will be adapted for each included database and/or information source. A hand search of the reference list of all included sources of evidence will be screened for additional studies.

### Data extraction

Data extraction will be performed using a pre-piloted data extraction form in Excel (Table 2.) [18].

**Table 2:** Data extraction form.

Date Form Completed:
Name/ID of Person:
ARTICLE NUMBER

**A: Bibliographical Information**
Title of paper, year of publication and authors
Year of publication
Country in which study was conducted
Study Funding Source
Possible conflicts of interest (for study authors)
Study Type (Cross sectional, Cohort, Systematic Review, Report)
Aim/purpose

**B: Population Characteristic**
Population Description (from which study participants are drawn)- Pregnant women in the first trimester
Source setting of the population (e.g., urban, rural, particular ethnic group)
Methods of recruitment of participants
Total number of participants (sample size)
Age

**C: Concept**
Main Category (urine metabolomics)
Sub Concept (preeclampsia)
Predictive capacity of urine glycolic acid and/or methyl succinic acid

**D: Outcomes of Interest**
Primary Outcomes as reported in the Study
Subgroup outcomes (ethnicity, age specific etc)
Other secondary outcome reported
Key Conclusions of Study authors

**Outcomes** The main outcome will be the development of preeclampsia, including early and late onset subtypes, as defined by the included studies.

### Risk of Bias Assessment

A risk of bias assessment will be done using the Quality in Prognosis Studies (QUIPS) tool [19]. The QUIPS tool will aid in the evaluation of the quality of methods of the included studies, including prognostic factor measurement, outcome measurement, confounders, statistical analysis, study participation and attrition and reporting. A sensitivity analysis will be done by excluding studies classified as having a ‘high’ overall risk of bias according to the QUIPS tool. The results from the primary analysis (including all studies) will be compared to the results from the sensitivity analysis and any differences will be noted and discussed.

### Data synthesis

Data synthesis will consist of a narrative of the trends of GA and MSA use for first-trimester preeclampsia risk stratification. Quantitative analysis maybe performed if more than three studies report comparable metrics. If applicable, meta-analysis will be performed using a random-effects model. Statistical analyses will be done using R and/or STATA. If meta-analysis is not feasible, forest plots of available adjusted estimates without calculated pooled summary effect will be presented. If fewer than three studies report eligible quantitative data, meta-analysis will not be done, instead a narrative synthesis will be performed to highlight existing gaps in literature and structure clear trajectory for future biomarker discoveries.

### Meta-bias(es) and confidence in cumulative evidence

We will assess publication-bias using funnel plots and Egger’s test if ≥10 studies are included. Selective reporting bias will be evaluated by comparing registered protocols with published results. The strength of evidence will be graded using the GRADE framework adapted for prognostic factor research, considering risk of bias (QUIPS), inconsistency, indirectness, imprecision, and publication bias [20].

### Ethical considerations

This review is a secondary analysis of peer-reviewed articles and is exempt from ethical approval.

### Dissemination plan

The findings will be published in a peer-reviewed journal and presented at conferences.

### Implications

This systematic review aims to evaluate the prognostic value and clinical utility of urinary glycolic acid and methylsuccinic acid for preeclampsia risk assessment in the first trimester. Urine represents an ideal non-invasive specimen for risk stratification, being easy to collect and store, even in remote areas. GA and MSA are compounds that could potentially be assayed cost-effectively. The findings from this review may inform the development of a low-cost risk-stratification tool for preeclampsia suitable for adoption in resource-limited settings.

### Strengths and limitations of this study

- This systematic review protocol details the methods for a thorough literature search on the available evidence on the use of first-trimester urinary glycolic acid and methylsuccinic acid as prognostic factors for preeclampsia development.
- The PRISMA checklist for systematic reviews will guide this review.
- Limiting the review to articles published in English may introduce language bias, as relevant studies in other languages could be missed.

### Protocol amendments

Any major protocol amendments will be documented in a table titled ‘Protocol Amendment Summary’. The table will include the following headings: date, protocol section affected, original text/plan, revised text/plan. See Table 3 for current changes to the protocol.

**Table 3:** Protocol amendment summary.

Date	Protocol section Affected	Original Text/Plan	Revised Text/Plan
06/07/2026	Justification/Introduction	Brief Rationale for choosing GA and MSA provided and unclear purpose of review.	More comprehensive justification given, review clearly positioned as a prognostic factor systematic review.
06/07/2026	Methods	Study timeline ending May 2026	Revised to end 31 August 2026
06/07/2026	Methods	Eligibility criteria not including comparator and timing (PICOT) and risk of bias assessment tools standard for prognostic factor studies.	PICOT added to the eligibility criteria and Risk of Bias tools adjusted to suit the standard for prognostic factor reviews (QUIPS).
06/07/2026	Search Strategy	Key words missing from the search terms.	Key words: ‘biomarkers’, ‘metabolomics’, ‘organic acids’ and ‘metabolite’ added to the search terms.

## Supporting information

S1 File PRISMA-P checklist completed for this study “PRISMA-P_Checklist_Completed. (DOCX)
