# Supplementary material for: First-trimester urinary glycolic acid and methylsuccinic acid measurement for preeclampsia prediction: A Systematic review protocol
Source: PLoS One. 2026 Sep 25;21(9):e0356735. doi: 10.1371/journal.pone.0356735 (PMC13614548; doi:10.1371/journal.pone.0356735)
Supplement: Supplementary file 1 [file pone.0356735.s001.docx]

# PRISMA-P 2015 Checklist — Completed

*Protocol reviewed: “First-Trimester Urinary Glycolic Acid and Methylsuccinic Acid Measurement for Preeclampsia Prediction: A Systematic Review Protocol” (PROSPERO ID* CRD420251057596*).*

*Adapted from Table 3 in Moher D et al. Preferred reporting items for systematic review and meta-analysis protocols (PRISMA-P) 2015 statement. Systematic Reviews 2015, 4:1.*

| **Section/topic** | **#** | **Checklist item** | **Reported** | **Location** |
| --- | --- | --- | --- | --- |
| Title | **1a** | Identify the report as a protocol of a systematic review | **Yes** | Page 1/line 4-5 |
| Title | **1b** | If an update of a previous review, identify as such | **No** | This is a de novo review. |
| Registration | **2** | If registered, provide registry name and registration number in the Abstract | **Yes** | Page 2/line 33-34 |
| Authors | **3a** | Provide name, institutional affiliation, and e-mail of all authors; physical mailing address of corresponding author | Yes | Page 1/line 7- 15 |
| Authors | **3b** | Describe author contributions and identify the guarantor | **Yes** | Page 1/ line 14 - 18 |
| Amendments | **4** | If an amendment of a completed/published protocol, identify and list changes; otherwise state the plan for documenting future amendments | Yes | Page 8/ line 213-214 |
| Support | **5a** | Indicate sources of financial or other support for the review | No | No funding received for this work. |
| Support | **5b** | Provide name of the review funder and/or sponsor | No |  |
| Support | **5c** | Describe role of funder(s)/sponsor(s) in developing the protocol | No |  |
| Rationale | **6** | Describe the rationale for the review in the context of what is already known | **Yes** | Page 3/ line 54-87 Introduction |
| Objectives | **7** | State the question(s) the review will address with reference to participants, interventions, comparators, and outcomes (PICO) | Yes | Page 3/ line 89 -90  Page 4/ line 108-113 |
| Eligibility criteria | **8** | Specify study and report characteristics (PICO, study design, setting, time frame, years, language, publication status) used as eligibility criteria | **Yes** | Page 4 / line 108-122 |
| Information sources | **9** | Describe all intended information sources with planned dates of coverage | **Yes** | Page 4/ line 97- 98 and Page 4-5/ line 124-128. |
| Search strategy | **10** | Present a draft search strategy for at least one database, with planned limits, so it could be repeated | **Yes** | Page 5 / line 137-138 |
| Data management | **11a** | Describe mechanism(s) for managing records and data throughout the review | **Yes** | Page 6 / line 144-151 |
| Selection process | **11b** | State the process for selecting studies through each phase (screening, eligibility, inclusion) | **Yes** | Page 6 / line 144-151 |
| Data collection process | **11c** | Describe planned method of extracting data (piloting, independent/duplicate extraction) and process for contacting authors | Yes | Page 6 / line 144 – 151 and Page 4 line 120-121 |
| Data items | **12** | List and define all variables for which data will be sought, including pre-planned assumptions/simplifications | **Yes** | Page 6 – 7/ line 155-156 |
| Outcomes and prioritization | **13** | List and define all outcomes sought, with prioritization of main vs. additional outcomes and rationale | **Yes** | Page 4/ line 111 |
| Risk of bias (individual studies) | **14** | Describe anticipated methods for assessing risk of bias; state how this will be used in data synthesis | Yes | Page 7 / line 160 - 166 |
| Synthesis | **15a** | Describe criteria under which study data will be quantitatively synthesized | **Yes** | Page 7/ line 168-176 |
| Synthesis | **15b** | Describe planned summary measures, methods of handling data, and exploration of consistency (e.g., I²) | Yes | Page 7 / line 168-176 |
| Synthesis | **15c** | Describe any proposed additional analyses (sensitivity, subgroup, meta-regression) | Yes | Page 7 / line 168-176 |
| Synthesis | **15d** | If quantitative synthesis is not appropriate, describe the type of summary planned | **Yes** | Page 7 / line 168-176 |
| Meta-bias(es) | **16** | Specify any planned assessment of meta-bias(es) (publication bias, selective reporting) | **Yes** | Page 7 / line 178-182 |
| Confidence in cumulative evidence | **17** | Describe how the strength of the body of evidence will be assessed (e.g., GRADE) | **Yes** | Page 7 / line 178-182 |
